# Strategies for Increasing Recruitment to Randomised Controlled Trials: Systematic Review

**DOI:** 10.1371/journal.pmed.1000368

**Published:** 2010-11-09

**Authors:** Patrina H. Y. Caldwell, Sana Hamilton, Alvin Tan, Jonathan C. Craig

**Affiliations:** 1 Centre for Kidney Research, The Children's Hospital at Westmead, New South Wales, Australia; 2 Discipline of Paediatrics and Child Health, University of Sydney, New South Wales, Australia; 3 The Children's Hospital at Westmead, New South Wales, Australia; 4 School of Public Health, University of Sydney, New South Wales, Australia; University Paris Descartes, France

## Abstract

**Background:**

Recruitment of participants into randomised controlled trials (RCTs) is critical for successful trial conduct. Although there have been two previous systematic reviews on related topics, the results (which identified specific interventions) were inconclusive and not generalizable. The aim of our study was to evaluate the relative effectiveness of recruitment strategies for participation in RCTs.

**Methods and Findings:**

A systematic review, using the PRISMA guideline for reporting of systematic reviews, that compared methods of recruiting individual study participants into an actual or mock RCT were included. We searched MEDLINE, Embase, The Cochrane Library, and reference lists of relevant studies. From over 16,000 titles or abstracts reviewed, 396 papers were retrieved and 37 studies were included, in which 18,812 of at least 59,354 people approached agreed to participate in a clinical RCT. Recruitment strategies were broadly divided into four groups: novel trial designs (eight studies), recruiter differences (eight studies), incentives (two studies), and provision of trial information (19 studies). Strategies that increased people's awareness of the health problem being studied (e.g., an interactive computer program [relative risk (RR) 1.48, 95% confidence interval (CI) 1.00–2.18], attendance at an education session [RR 1.14, 95% CI 1.01–1.28], addition of a health questionnaire [RR 1.37, 95% CI 1.14–1.66]), or a video about the health condition (RR 1.75, 95% CI 1.11–2.74), and also monetary incentives (RR1.39, 95% CI 1.13–1.64 to RR 1.53, 95% CI 1.28–1.84) improved recruitment. Increasing patients' understanding of the trial process, recruiter differences, and various methods of randomisation and consent design did not show a difference in recruitment. Consent rates were also higher for nonblinded trial design, but differential loss to follow up between groups may jeopardise the study findings. The study's main limitation was the necessity of modifying the search strategy with subsequent search updates because of changes in MEDLINE definitions. The abstracts of previous versions of this systematic review were published in 2002 and 2007.

**Conclusion:**

Recruitment strategies that focus on increasing potential participants' awareness of the health problem being studied, its potential impact on their health, and their engagement in the learning process appeared to increase recruitment to clinical studies. Further trials of recruitment strategies that target engaging participants to increase their awareness of the health problems being studied and the potential impact on their health may confirm this hypothesis.

*Please see later in the article for the Editors' Summary*

## Abstract 2

Patrina Caldwell and colleagues performed a systematic review of randomized studies that compared methods of recruiting individual study participants into trials, and found that strategies that focus on increasing potential participants' awareness of the specific health problem, and that engaged them, appeared to increase recruitment.

## Editors' Summary

**Background:**

Before any health care intervention—a treatment for a disease or a measure such as vaccination that is designed to prevent an illness—is adopted by the medical community, it undergoes exhaustive laboratory-based and clinical research. In the laboratory, scientists investigate the causes of diseases, identify potential new treatments or preventive methods, and test these interventions in animals. New interventions that look hopeful are then investigated in clinical trials—studies that test these interventions in people by following a strict trial protocol or action plan. Phase I trials test interventions in a few healthy volunteers or patients to evaluate their safety and to identify possible side effects. In phase II trials, a larger group of patients receives an intervention to evaluate its safety further and to get an initial idea of its effectiveness. In phase III trials, very large groups of patients (sometimes in excess of a thousand people) are randomly assigned to receive the new intervention or an established intervention or placebo (dummy intervention). These “randomized controlled trials” or “RCTs” provide the most reliable information about the effectiveness and safety of health care interventions.

**Why Was This Study Done?:**

Patients who participate in clinical trials must fulfill the inclusion criteria laid down in the trial protocol and must be given information about the trial, its risks, and potential benefits before agreeing to participate (informed consent). Unfortunately, many RCTs struggle to enroll the number of patients specified in their trial protocol, which can reduce a trial's ability to measure the effect of a new intervention. Inadequate recruitment can also increase costs and, in the worst cases, prevent trial completion. Several strategies have been developed to improve recruitment but it is not clear which strategy works best. In this study, the researchers undertake a systematic review (a study that uses predefined criteria to identify all the research on a given topic) of “recruitment trials”—studies that have randomly divided potential RCT participants into groups, applied different strategies for recruitment to each group, and compared recruitment rates in the groups.

**What Did the Researchers Do and Find?:**

The researchers identified 37 randomized trials of recruitment strategies into real and mock RCTs (where no actual trial occurred). In all, 18,812 people agreed to participate in an RCT in these recruitment trials out of at least 59,354 people approached. Some of these trials investigated novel strategies for recruitment, such as changes in how patients are randomized. Others looked at the effect of recruiter differences (for example, increased contact between the health care professionals doing the recruiting and the trial investigators), the effect of offering monetary incentives to participants, and the effect of giving more information about the trial to potential participants. Recruitment strategies that improved people's awareness of the health problem being studied—provision of an interactive computer program or a video about the health condition, attendance at an educational session, or inclusion of a health questionnaire in the recruitment process—improved recruitment rates, as did monetary incentives. Increasing patients' understanding about the trial process itself, recruiter differences, and alterations in consent design and randomization generally had no effect on recruitment rates although consent rates were higher when patients knew the treatment to which they had been randomly allocated before consenting. However, differential losses among the patients in different treatment groups in such nonblinded trials may jeopardize study findings.

**What Do These Findings Mean?:**

These findings suggest that trial recruitment strategies that focus on increasing the awareness of potential participants of the health problem being studied and its possible effects on their health, and that engage potential participants in the trial process are likely to increase recruitment to RCTs. The accuracy of these findings depends on whether the researchers identified all the published research on recruitment strategies and on whether other research on recruitment strategies has been undertaken and not published that could alter these findings. Furthermore, because about half of the recruitment trials identified by the researchers were undertaken in the US, the successful strategies identified here might not be generalizable to other countries. Nevertheless, these recruitment strategies should now be investigated further to ensure that the future evaluation of new health care interventions is not hampered by poor recruitment into RCTs.

**Additional Information:**

Please access these Web sites via the online version of this summary at http://dx.doi.org/10.1371/journal.pmed.1000368.

- The ClinicalTrials.gov Web site is a searchable register of federally and privately supported clinical trials in the US and around the world, providing information about all aspects of clinical trials
- The US National Institutes of Health provides information about clinical trials
- The UK National Health Service Choices Web site has information for patients about clinical trials and medical research
- The UK Medical Research Council Clinical Trials Units also provides information for patients about clinical trials and links to information on clinical trials provided by other organizations
- MedlinePlus has links to further resources on clinical trials (in English and Spanish)
- The Australian Government's National Health and Medical Research Council has information about clinical trials
- WHO International Clinical Trials Registry Platform aims to ensure that all trials are publicly accessible to those making health care decisions
- The Star Child Health International Forum of Standards for Research is a resource center for pediatric clinical trial design, conduct, and reporting

## Introduction

The randomised controlled trial (RCT) provides the most reliable evidence for evaluating the effects of health care interventions [1],[2], but the successful conduct of clinical RCTs is often hindered by recruitment difficulties [3]. Inadequate recruitment reduces the power of studies to detect significant intervention effects [4], causes delays (which may affect the generalizability of the study if standard care changes over time), increases costs, and can lead to failure to complete trials [5],[6]. With increasing reliance on clinical RCT findings for clinical and regulatory decision making, the success of future RCTs depends on employing effective and efficient methods for recruiting study participants [7].

Historically recruitment of participants for RCTs has been by “trial and error” [8], by using a number of different strategies and modifying strategies according to the observed effects on recruitment. More recently, novel strategies have been developed to facilitate adequate and timely recruitment [3],[4]. Although there have been two previous systematic reviews on strategies to enhance recruitment to research [9],[10], they identified specific individual interventions. However, these interventions could not be combined to offer useful general advice for recruitment for clinical RCTs.

The aim of this study was to identify effective recruitment strategies for clinical RCTs by systematically reviewing randomised studies that compare consent rates, or other methods of measuring consent for two or more recruitment methods used, to approach potential RCT participants for trial participation (these studies are termed recruitment trials).

## Methods

A protocol for this systematic review had not been registered before the review commenced, although the abstracts of previous versions of this systematic review were published in 2002 (International Clinical Trials Symposium: improving health care in the new millennium) [11] and 2007 (3rd International Clinical Trials Symposium) [12] (Text S1).

### Selection Criteria

All randomised and quasi-randomised studies that compared two or more methods of recruiting study participants to a real phase III RCT or mock RCT (where no actual trial occurred) were included. Studies that assessed recruitment to observational studies, questionnaires, health promotional activities, and other health care interventions and nonrandomised studies of recruitment strategies were excluded. Where more than one publication of the same study existed, the publication with the most complete data was included.

### Literature Search

**Figure 1:**
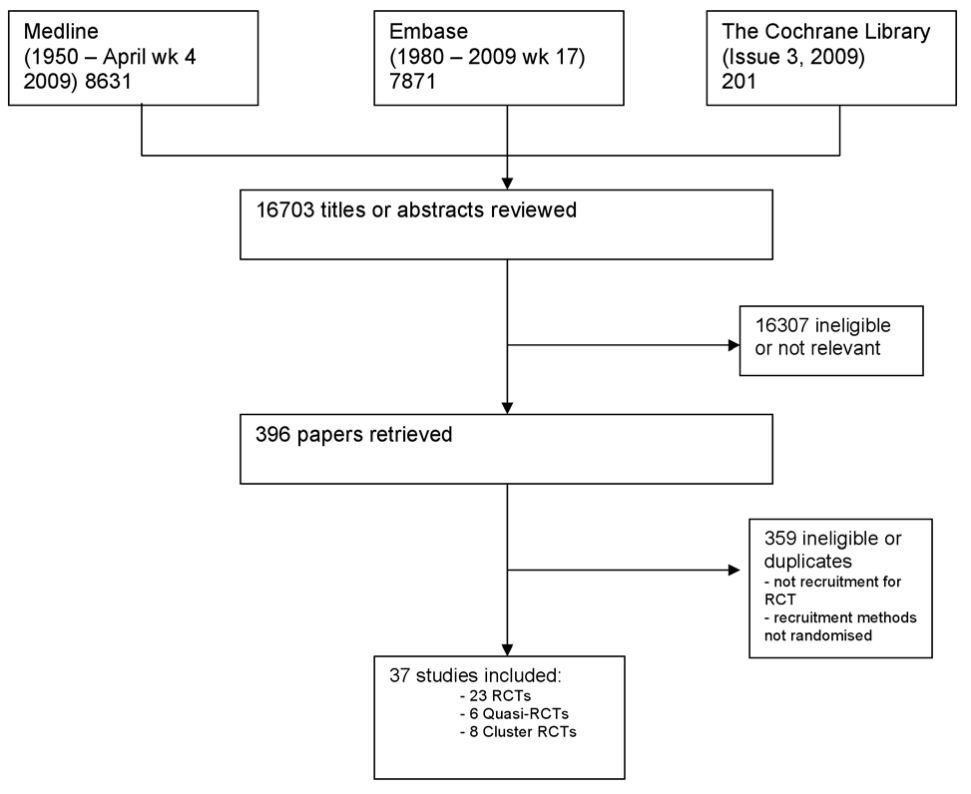
Literature search.

Studies were identified from MEDLINE (1950 to April, week 4, 2009), Embase (1980 to week 17, 2009), and The Cochrane Library (Cochrane Library, issue 3, 2009) (Figure 1). The MEDLINE and Embase databases were searched using text words and subject headings (with unlimited truncations) for “recruitment,” “enrolment,” and “accrual” combined with “random” and “trials” and “participate” or “consent” or “recruit” with unlimited truncations. The Cochrane Library was searched using “recruitment” combined with “random and trial,” and “consent or accrual.” The search strategy changed slightly with time as a result of changes in MEDLINE Mesh heading definitions. Reference lists of relevant studies were also searched and non-English language papers were translated. Two of three reviewers (PHYC, AT, or SH) independently screened each study title and abstract for eligibility, retrieved full text articles of all potentially relevant studies, and extracted data from the retrieved papers using a form that was designed by the authors. Disagreements were resolved by discussion with a third reviewer (JCC).

### Data Extraction

Data were extracted without blinding to authorship, on the recruitment methods evaluated, the population setting, and the trial design, as well as risk of bias items such as randomisation, allocation concealment, blinding of outcome assessors, loss to follow up, and intention-to-treat analysis. These elements were each assessed separately using the method developed by the Cochrane Collaboration [13].

### Outcomes Assessed

**Figure 2:**
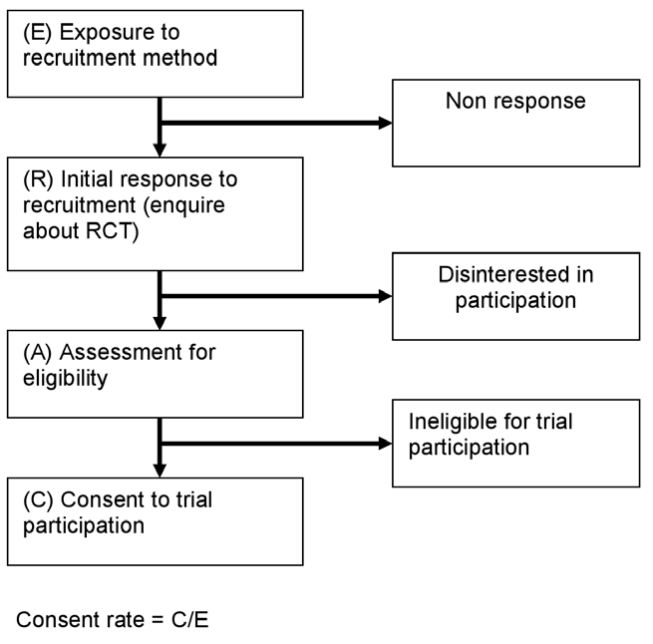
Consent rate for RCTs.

The primary outcome of interest was consent rates for the different recruitment strategies. Because studies differed in definitions of consent rates, where possible we recalculated the consent rate of each recruitment method by dividing the number of participants exposed to the recruitment method who actually consented for clinical study participation by the total number of potential participants exposed to that method (see Figure 2). For studies where information was insufficient to calculate consent rates, other measures of consent success described in the study were reported. For mock trials, willingness to consent to participate (i.e., potential participants acknowledging that they would be willing to participate in the trial or willingness to be contacted for participation in future trials) was the outcome measure. Consent rates and other outcome measures were compared using intention-to-treat analysis.

### Statistical Methods

Where possible we used relative risk (RR) and their 95% confidence intervals (CIs) to describe the effects of different strategies in individual recruitment trials. Where more than two strategies were used in a single recruitment trial, the numerator and denominator from the standard (control) recruitment strategy was divided by the number of intervention strategies for each comparison so that the control numbers would not be overrepresented [13].

## Results

### Literature Search

From 16,703 unique titles and abstracts, 396 articles were retrieved and 37 eligible publications identified (Figure 1). Collectively this total assessed recruitment outcomes in at least 59,354 people who were approached for clinical study participation, of whom 18,812 consented to participate (Table 1). (Not all studies identified the number of potential participants who were approached).

**Table 1:** Included studies.

Trial Type	Author	Year of Publication	Country of Trial	Health Problem Studied	Intervention Arms of RCT	Recruitment Strategy Studied	*n* Recruited for Trial	*n* Invited to Participate in Trial
Treatment	Du [39]	2008	USA	Lung cancer	Mixed treatments (multiple trials)	Information provision	26	126
	Hutchison [38]	2007	UK	Multiple cancers	Mixed treatments (multiple trials)	Information provision	128	173
	Monaghan [51]	2006	Multinational	BP control in diabetics	Antihypertensive versus placebo	Recruiter differences	7,847	167 sites
	Litchfield [42] a	2005	UK	Diabetes	Two insulin delivery systems	Recruiter differences	73	80
	Kimmick [40]	2005	USA	Multiple cancers	Mixed treatments (multiple trials)	Recruiter differences	1,097	unknown
	Nystuen [31]	2004	Norway	Absentee employees	Follow up versus standard care	Information provision	97	703
	Donovan [23]	2003	UK	Prostate cancer	Surgery versus radiotherapy versus monitoring	Recruiter differences	103	150
	Coyne [48]	2003	USA	Multiple cancers	Chemotherapy (multiple trials)	Information provision	147	226
	Quinaux [41]	2003	France	Breast cancer	Chemotherapies	Recruiter differences	362	unknown
	Tworoger [37]	2002	USA	Breast cancer	Aerobic exercises versus stretching	Information provision	376	4,999
	Fleissig [49]	2001	UK	Multiple cancers	Mixed treatments (multiple trials)	Recruiter differences	205	265 (15 recruiters)
	Miller [43]	1999	USA	Depression	Psychotherapy versus antidepressants versus both	Recruiter differences	50	347
	Cooper [22]	1997	UK	Menorrhagia	Medical management versus surgery	Trial design	187	273
	Berner [45]	1997	USA	Gynaecological cancers	Mixed treatments (multiple trials)	Information provision	9	120
	Aaronson [20]	1996	The Netherlands	Multiple cancers	Chemotherapy (multiple trials)	Information provision	146	346
	Wadland [35]	1990	USA	Smoking	Nicotine gum versus standard care	Information provision	52	104
	Simes [33]	1986	Australia	Multiple cancers	Mixed treatments (multiple trials)	Information provision	50	57
Prevention	Leira [29]	2009	USA	Aspiration pneumonia	Ranitidine versus placebo	Information provision	52	100
	Mandelblatt [3] a	2005	USA	Breast cancer	Tamoxifen versus Raloxifene	Information provision	325	450
	Avenell [21] a	2004	UK	Fractures	Vitamins versus placebo/no treatment	Trial design	367	538
	Ford [25]	2004	USA	Multiple cancers	Screening tests versus standard care	Information provision	376	12,400
	Hemminki [27] a	2004	Estonia	Postmenopausal health risks	Hormone replacement versus placebo/ no treatment	Trial design	1,823	4,295
	Larkey [50]	2002	USA	cardiovascular disease, cancer and osteoporosis	Hormone replacement therapy and dietary modification and calcium and vitamin D supplements	Recruiter differences	13	34+
	Kendrick [4]	2001	UK	Home safety	Safety equipment versus usual care	Information provision	374	2,397
	Kiernan [28]	2000	USA	Healthy diet	Additional goal setting techniques versus standard care	Information provision	9	561
	Welton [46] a	1999	UK	menopausal symptoms and osteoporosis	Hormone replacement therapies versus placebo	Trial design	150	492 (438)
	Rogers [32]	1998	USA	Risk for life threatening illness	Follow up versus standard care	Trial design	44	57
	Valanis [34]	1998	USA	Lung cancer	Vitamins versus placebo	Information provision	451	22,546
Mock trial	Halpern [47]	2004	USA	Hypertension	Different hypertensives	Incentives+trial design	66–94	142
	Ellis [24]	2002	Australia	Breast cancer	Chemotherapy versus Tamoxifen	Information provision	26	180
	Martinson [6] a	2000	USA	Smoking cessation and prevention	Peer, mail, and phone contacts versus standard care	Incentives	1,560	4,046
	Wragg [44]	2000	UK	Postmenopausal health risks	Hormone replacement versus placebo	Information provision	22	50
	Myles [30]	1999	Australia	Anaesthesia for surgery	Experimental drug versus standard care	Trial design	429	770
	Weston [5] a	1997	Canada	Premature labour	Induced labour versus expectant management	Information provision	43	90
	Gallo [26] a	1995	Italy	Hypothetical disease	Experimental drug versus standard drug	Trial design	1,620	2,035
	Llewellyn-Thomas [2] a	1995	Canada	Bowel cancer	Chemotherapy versus monitoring	Information provision	52	102
	Simel [36] a	1991	USA	Variable presenting health problems	Standard versus new medication	Trial design	55	100
Total							18,812	59,354+

a Studies showed a statistically significant difference in consent rates between recruitment strategies.

BP, blood pressure.

### Quality of Included Studies

There were 23 parallel group RCTs, six quasi-RCTs (including one using paired data), and eight cluster RCTs. Of the 37 included recruitment trials, only 12 studies (32%) had clear allocation concealment, two (4%) specified blinding of outcome assessors (no study had blinding of participants as this would have been difficult to achieve), 15 (40%) recorded loss to follow-up information, and 14 (38%) used intention-to-treat analysis (see Table 2).

**Table 2:** Quality of included studies.

Trial Type	Author	Type Of RCT	Allocation Concealment	Blinding of Outcome Assessors	Loss to Follow Up Mentioned	Intention-to-Treat Analysis	Quality Items
Prevention	Avenell [21]	Parallel	Yes	No	Yes	Yes	3
Prevention	Rogers [32]	Parallel	Yes	Yes	No	Yes	3
Treatment	Monaghan [51]	Cluster RCT	Yes	Unclear	Unclear	Yes	2
Treatment	Hutchison [38]	Parallel	Yes	Unclear	Unclear	Yes	2
Treatment	Cooper [22]	Parallel	Yes	No	No	Yes	2
Treatment	Tworoger [37]	Parallel	Unclear	Unclear	Yes	Yes	2
Treatment	Coyne [48]	Cluster RCT	Unclear	No	Yes	Yes	2
Treatment	Du [39]	Parallel	Unclear	Yes	Yes	No	2
Prevention	Kendrick [4]	Parallel	Yes	No	Yes	Unclear	2
Prevention	Hemminki [27]	Parallel	Yes	No	Unclear	Yes	2
Prevention	Ford [25]	Parallel	Unclear	No	Yes	Yes	2
Prevention	Leira [29]	Parallel	No	Unclear	Yes	Yes	2
Mock trial	Weston [5]	Parallel	Yes	No	Yes	Unclear	2
Mock trial	Ellis [24]	Parallel	Yes	No	Yes	Unclear	2
Mock trial	Llewellyn-Thomas [2]	Parallel	Yes	No	Yes	No	2
Mock trial	Martinson [6]	Cluster RCT	Yes	No	Unclear	Yes	2
Treatment	Donovan [23]	Parallel	Yes	No	No	No	1
Treatment	Wadland [35]	Parallel	Unclear	No	Yes	Unclear	1
Treatment	Aaronson [20]	Parallel	Unclear	No	Yes	Unclear	1
Treatment	Berner [45]	Quasi-RCT	No	Unclear	Yes	Unclear	1
Treatment	Nystuen [31]	Parallel	No	Unclear	Unclear	Yes	1
Prevention	Larkey [50]	Cluster RCT	Unclear	No	Yes	No	1
Prevention	Valanis [34]	Parallel	Unclear	No	No	Yes	1
Prevention	Welton [46]	Quasi-RCT	No	No	Yes	Unclear	1
Mock trial	Simel [36]	Parallel	Unclear	No	No	Yes	1
Treatment	Quinaux [41]	Cluster RCT	Unclear	Unclear	Unclear	Unclear	0
Treatment	Kimmick [40]	Cluster RCT	Unclear	Unclear	Unclear	Unclear	0
Treatment	Litchfield [42]	Cluster RCT	Unclear	Unclear	Unclear	Unclear	0
Treatment	Fleissig [49]	Cluster RCT	Unclear	No	No	Unclear	0
Treatment	Simes [33]	Parallel	No	No	No	Unclear	0
Treatment	Miller [43]	Quasi-RCT	No	No	No	Unclear	0
Prevention	Kiernan [28]	Parallel	Unclear	No	No	Unclear	0
Prevention	Mandelblatt [3]	Quasi-RCT	No	No	Unclear	Unclear	0
Mock trial	Gallo [26]	Parallel	Unclear	No	No	Unclear	0
Mock trial	Myles [30]	Parallel	Unclear	No	No	Unclear	0
Mock trial	Wragg [44]	Quasi-RCT	Unclear	No	No	Unclear	0
Mock trial	Halpern [47]	Paired data	No	No	Unclear	Unclear	0

### Characteristics of Included Studies

Of the 37 included studies, 17 assessed treatment comparisons, 11 were prevention studies, and nine mock studies (where participants declared their willingness to participate in a trial but no actual trial occurred).

There were 66 different types of recruitment strategies that were broadly categorised into four groups: novel trial designs (nine studies), recruiter differences (eight studies), incentives (two studies), and provision of trial information (19 studies), with one study looking at both novel trial design and incentives [14]. Standard recruitment is defined as when the investigator invites the potential participant to enrol in the study and treatment allocation is randomly assigned after consent has been given, with routine treatment being provided where consent is not given.

### Types of Recruitment Strategies Studied

#### Novel trial designs

**Figure 3:**
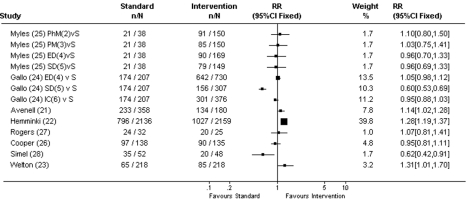
Consent rates for novel trial designs. RR, intervention recruitment strategy/standard recruitment strategy. Used total number/number of intervention strategies to calculate RR, so that the number of patients on standard strategies were not overrepresented; S, random assignment for participants, standard care for nonparticipants; 2, patients are told physician believes the experimental drug may be superior. Increased chance of receiving the experimental drug after consenting; 3, patients are told that they are allowed to increase or decrease their chance of receiving the new experimental drug after consenting; 4, experimental drug for participants, standard care for nonparticipants; 5, standard drug for participants, experimental drug for nonparticipants; 6, random assignment for participants, choice of either treatments for nonparticipants.

Avenell and Hemminki [15],[16] compared a standard placebo-controlled design with a nonblinded trial design (both for prevention studies) (see Figure 3 and Table 3). In the nonblinded trial design arm, randomisation occurred before participants were approached, and participants were informed of the treatment they were randomised to receive prior to giving consent. Consent rates were higher for the nonblinded trial design compared with standard trial design where randomisation occurred after consent for trial participation (RR 1.14, 95% CI 1.02–1.28 and RR 1.28, 95% CI 1.19–1.37, respectively) [15],[16]. Welton [17] compared a noninferiority clinical study (where both arms of the trial had an active treatment) with a placebo-controlled study of hormone replacement for postmenopausal women. Willingness to enrol in the clinical study appeared to be higher for the noninferiority study compared with the placebo-controlled study, although results were only just statistically significant (39% versus 30%, RR 1.31, 95% CI 1.01–1.70).

**Table 3:** Studies of novel trial designs.

Study	Standard Recruitment Strategy	*n/N*	Consent Rate (95% CI)	Experimental Recruitment Strategies	*n/N*	Consent Rate (95% CI)	RR (95% CI)
Myles [30]	One-sided informed consent^a^	84/151	56% (48–64)	One-sided physician modified^b^	91/150	61% (52–69)	1.10 (0.80–1.50)
				One-sided patient modified^c^	85/150	57% (48–65)	1.03 (0.75–1.41)
				Prerandomised to experimental drug^d^	90/169	53% (45–61)	0.96 (0.70–1.33)
				Prerandomised to standard drug^e^	79/149	53% (45–61)	0.96 (0.69–1.33)
Gallo [26]	One-sided informed consent^a^	521/622	84% (81–87)	Prerandomised to experimental drug^d^	642/730	88% (86–90)	1.05 (0.98–1.12)
				Prerandomised to standard drug^e^	156/307	51% (45–56)	0.60 (0.53–0.69)^f^
				Two-sided informed consent^g^	301/376	80% (76–84)	0.95 (0.88–1.03)
Avenell [21]	Standard placebo-controlled design	233/358	65% (60–70)	Nonblinded trial design	134/180	74% (67–81)	1.14 (1.02–1.28)^f^
Hemminki [27]	Standard placebo-controlled design	796/2,136	37% (35–39)	Nonblinded trial design	1,027/2159	48% (46–50)	1.28 (1.19–1.37)^f^
Rogers [32]	Opting-in consent for participation	24/32	75% (57–89)	Opting-out consent for nonparticipation	20/25	80% (59–93)	1.07 (0.81–1.41)
Cooper [22]	Standard informed consent	97/138	70% (62–78)	Partially randomised patient preference^h^	90/135	67% (58–75)	0.95 (0.81–1.11)
Simel [36]	Consent for trial of usual treatment versus new treatment that may work twice as fast	35/52	67% (53–80)	Consent for trial of usual treatment versus new treatment that may work half as fast	20/48	41% (28–57)	0.62 (0.42–0.91)^f^
Halpern [47] A- US$100 incentive	10% risk of adverse effects	26/64	41% (29–54)	20% risk of adverse effects	23/64	36% (24–49)	1.08 (0.59–2.00)
	10% risk of adverse effects	26/64	41% (29–54)	30% risk of adverse effects	18/64	28% (18–41)	1.44 (0.72–2.89)
	20% risk of adverse effects	23/64	36% (24–49)	30% risk of adverse effects	18/64	28% (18–41)	1.33 (0.65–2.72)
Halpern [47] A- US$1,000 incentive	10% risk of adverse effects	33/64	52% (39–64)	20% risk of adverse effects	26/64	41% (29–54)	1.31 (0.77–2.22)
	10% risk of adverse effects	33/64	52% (39–64)	30% risk of adverse effects	23/64	36% (24–49)	1.42 (0.81–2.46)
	20% risk of adverse effects	26/64	41% (29–54)	30% risk of adverse effects	23/64	36% (24–49)	1.08 (0.59–2.00)
Halpern [47] A- US$2,000 incentive	10% risk of adverse effects	35/64	55% (42–67)	20% risk of adverse effects	29/64	45% (33–58)	1.20 (0.74–1.94)
	10% risk of adverse effects	35/64	55% (42–67)	30% risk of adverse effects	25/64	39% (27–52)	1.38 (0.82–2.33)
	20% risk of adverse effects	29/64	45% (33–58)	30% risk of adverse effects	25/64	39% (27–52)	1.15 (0.66–2.02)
Halpern [47] B- US$100 incentive	10% assigned to placebo	21/62	34% (22–47)	30% assigned to placebo	20/62	32% (21–45)	1.10 (0.55–2.21)
	10% assigned to placebo	21/62	34% (22–47)	50% assigned to placebo	19/62	31% (20–44)	1.10 (0.55–2.21)
	30% assigned to placebo	20/62	32% (21–45)	50% assigned to placebo	19/62	31% (20–44)	1.00 (0.49–2.06)
Halpern [47] B- US$1,000 incentive	10% assigned to placebo	27/62	44% (31–57)	30% assigned to placebo	25/62	40% (28–54)	1.08 (0.61–1.90)
	10% assigned to placebo	27/62	44% (31–57)	50% assigned to placebo	23/62	37% (25–50)	1.17 (0.65–2.10)
	30% assigned to placebo	25/62	40% (28–54)	50% assigned to placebo	23/62	37% (25–50)	1.08 (0.59–1.99)
Halpern [47] B- US$2,000 incentive	10% assigned to placebo	28/62	45% (33–58)	30% assigned to placebo	26/62	42% (30–55)	1.08 (0.61–1.90)
	10% assigned to placebo	28/62	45% (33–58)	50% assigned to placebo	27/62	44% (31–57)	1.00 (0.58–1.73)
	30% assigned to placebo	26/62	42% (30–55)	50% assigned to placebo	27/62	44% (31–57)	0.93 (0.53–1.64)
Welton [46]	Standard placebo-controlled design	65/218	30% (24–36)	Noninferiority trial design	85/218	39% (33–46)	1.31 (1.01–1.70)^f^

RR, experimental recruitment strategy/standard recruitment strategy. Used total number/number of experimental strategies to calculate RR, so that standard was not overrepresented. Halpern's study used each participant more than once.

a Random assignment for participants, standard care for nonparticipants.

b Patients told physician believes the experimental drug may be superior. Increased chance of receiving the experimental drug after consenting.

c Patients are told that they are allowed to increase or decrease their chance of receiving the new experimental drug after consenting.

d Experimental drug for participants, standard care for nonparticipants.

e Standard drug for participants, experimental drug for nonparticipants.

f Studies showed a statistically significant difference in consent rates between recruitment strategies.

g Random assignment for participants, choice of either treatments for nonparticipants.

h Patients could choose to be randomised or choose their own treatment, but only those who chose to be randomised were compared with standard treatment.

Gallo and Myles (both for mock studies) compared standard randomisation (random assignment for all participants and standard care for nonparticipants) with different types of randomisation designs [18],[19]. Strategies included increasing or decreasing the chance of receiving the experimental treatment; experimental treatment for all participants and standard treatment for nonparticipants (where potential participants are informed that they have been randomised to receive the experimental treatment, but if they do not consent, they would receive the standard treatment); standard care for all participants and experimental treatment for nonparticipants (where potential participants are informed that they have been randomised to receive the standard treatment, but if they do not consent, they would receive the experimental treatment); and random assignment of treatment for participants and choice of treatment for nonparticipants. The only randomisation strategy that influenced consent was the “prerandomisation to standard drug” (standard care for all participants and experimental treatment for nonparticipants) in Gallo's study [18], which significantly reduced the consent rate compared with standard randomisation (RR 0.60, 95% CI 0.53–0.69) [18]. However, this was not demonstrated in Myles' study [19].

Cooper compared standard consent with partially randomised patient preference where patients could choose to be randomised or choose their own (medical or surgical) treatment [20]. Patients who chose their own treatment were excluded in our analysis, as choice of treatment conflicts with the purposes of random allocation of treatment, and only patients who chose to be randomised were compared with those receiving standard RCT consent (where they were offered the opportunity to participate in a clinical study where treatment was randomly allocated for participants). This study tested whether allowing a patient choice of treatments increased consent for choosing to have their treatment randomised, compared with simply inviting them to participate in a clinical RCT (without mentioning choice of treatment). There was no difference in consent rates between the standard consent and choosing to be randomised (RR 0.95, 95% CI 0.81–1.11).

Rogers compared “opting in” with “opting out” [21] where consent was sought for participation or for nonparticipation, respectively. In the “opting out” arm, consent rate for clinical study participation was calculated as the proportion who did not sign the consent form (for refusing participation). There was no difference in consent rates between the two groups (RR 1.07, 95% CI 0.81–1.41).

Simel compared consenting to a clinical study assessing standard medication versus a new medication that worked twice as fast with a clinical study comparing standard medication with a new medication that worked half as fast as the standard medication [22]. Participants were not informed that this was a mock trial. This study was designed to assess patients' competence and judgement regarding clinical study participation. Not surprisingly, more patients consented to a clinical study comparing the faster new medication than to a clinical study comparing a slower new medication (67% versus 41%, RR 0.62, 95% CI 0.42–0.91), with a more marked difference among those who voluntarily mentioned the medication's speed of action as a factor in their decision regarding clinical study participation, which may reflect better understanding of the trial information.

Halpern [14] used a factorial design to assess willingness to participate in a number of mock trials using paired data from the same individuals with variations in clinical study designs (as well as variation in monetary incentives, which will be discussed later under “incentives”). There were no differences in consent rates statistically.

#### Recruiter differences

**Figure 4:**
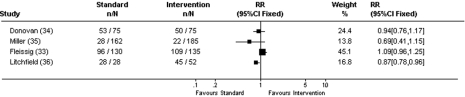
Consent rates for recruiter differences. RR, intervention recruitment strategy/standard recruitment strategy.

Eight recruitment trials compared recruiter differences (see Figure 4 and Table 4). Three cluster RCTs compared different strategies for engaging recruiters (e.g., standard contact versus additional monitoring and contact with recruiters [23]–[25]). Outcome measures were different for each of the studies and therefore results could not be combined. In Quinaux's study, 186 patients from 34 control centres enrolled compared with 176 total patients from 34 monitored centres [23]. In Kimmick's study, 1,161 elderly patients (36% of total patients in first year and 31% in second year) from the control centres enrolled compared with 1,075 (32% in first year and 31% in second year) from the centres who received additional training and contact with investigators [24]. Monaghan's study assessed median number of patients recruited per site with 37.0 patients from the 82 control sites compared with 37.5 patients from the 85 sites with increased contacts with investigators [25]. In all three studies, increased contact with investigators did not statistically increase consent rates, and appeared to actually lower enrolment. One recruitment trial that compared untrained recruiters with training of recruiters [26] found statistically more patients enrolled when the recruiter was trained (28 trained recruiters enrolled 13 patients versus 28 untrained recruiters who enrolled no patients). Fleissig compared standard recruitment with providing recruiters with information about patient preferences [27], with no differences in consent rates between the two methods (RR 1.09, 95% CI 0.96–1.25).

**Table 4:** Studies of recruiter differences.

Study	Standard Recruitment Strategy	*n/N*	Consent Rate (95% CI)	Experimental Recruitment Strategies	*n/N*	Consent Rate (95% CI)	RR (95% CI)
Donovan [23]	Recruitment by urologist	53/75	71% (59–81)	Recruitment by nurse	50/75	67% (55–77)	0.94 (0.76–1.17)
Miller [43]	Recruitment by senior investigator	28/162	17% (12–24)	Recruitment by research assistant	22/185	12% (8–17)	0.69 (0.41–1.15)
Fleissig [49]	Standard consent, doctors not aware of patients' personal preferences	96/130	74% (65–81)	Doctors shown patient's responses to questionnaire regarding personal preferences and trial participation before recruiting patients for trial	109/135	81% (73–87)	1.09 (0.96–1.25)
Litchfield [42]	Paper-based data recording	28/28 screened	100% (88–100)	Internet data capture	45/52 screened	87% (74–94)	0.87 (0.78–0.96)^a^
Quinaux [41]	Centres not monitored	186/34 centres		Monitored centres	176/34 centres		
Larkey [50]	Recruiters not trained	0/28 recruiters		Recruiters trained	13/28 recruiters		
Kimmick [40]	Standard recruitment, website access and periodic notification	777 (year 1)+384 (year 2) = 1,161		Additional seminar, educational materials, list of available protocols, email and mail reminders, and case discussion seminars for recruiters	691 year 1)+384 (year 2) = 1,075		
Monaghan [51]	Usual communication	37 (median) per site at 82 sites		Frequent email contact and individual feedback about recruitment to the recruiter	37.5 (median) per site at 85 sites		

RR, experimental recruitment strategy/standard recruitment strategy.

a Studies showed a statistically significant difference in consent rates between recruitment strategies.

Donovan and Miller compared recruiter roles (doctor versus nurse RR 0.94, 95% CI 0.76–1.17 [28], and senior investigator versus research assistant RR 0.69, 95% CI 0.41–1.15 [29]). Although there was no difference in consent rates between the recruiters, costs were higher for the more senior person (mean cost of £43.29 versus £36.40 and US$78.48 versus US$50.28 per patient randomised, respectively).

Litchfield compared internet-derived database handling with paper-based database handling [30]. Although proportionately more patients enrolled with the paper-based database, the internet database was more efficient (with shorter time required for data collection and more patients being exposed to the trial). 100% of paper-based database versus 87% internet database groups enrolled (RR 0.87, 95% CI 0.78–0.96), with the internet database being preferable for recruiters.

#### Incentives

**Figure 5:**
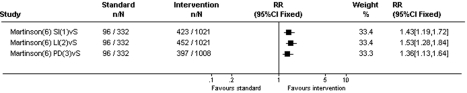
Consent rates for incentives. RR, intervention recruitment strategy/standard recruitment strategy. Used total number/number of intervention strategies to calculate RR, so that the number of patients on standard strategies were not overrepresented; S, random assignment for participants, standard care for nonparticipants; 1, small incentives (US$2 prepaid cash incentive); 2, larger incentive (US$15) contingent on response; 3, US$200 prize draw.

Martinson and Halpern assessed incentives for increasing recruitment (see Figure 5 and Table 5) [14],[31]. In the Martinson study, compared to no incentives, any monetary incentive increased survey response rates and willingness to be contacted regarding a smoking cessation trial. The study did not measure actual recruitment to the clinical study. Consent rate for no incentives was 29% compared with 41% for prepaid US$2 cash incentive (RR 1.43, 95% CI 1.19–1.72); 44% for US$15 cash incentive contingent on completion of survey (RR 1.53, 95% CI 1.28–1.84); and 39% for US$200 prize draw (RR 1.36, 95% CI 1.13–1.64).

**Table 5:** Studies of incentives.

Study	Standard Recruitment Strategy	*n/N*	Consent Rate (95% CI)	Experimental Recruitment Strategies	*n/N*	Consent Rate (95% CI)	RR (95% CI)
Martinson [6]	No incentives	288/996	29% (26–32)	US$2 small prepaid cash	423/1,021	41% (38–45)	1.43 (1.19–1.72)^a^
				Large cash incentives contingent on response (US$15)	452/1,021	44% (41–47)	1.53 (1.28–1.84)^a^
				US$200 prize draw	397/1008	39% (36–42)	1.36 (1.13–1.64)^a^
Halpern [47] A-10% risk of adverse effect	US$100	26/64	41% (29–54)	US$1,000	33/64	52% (39–64)	0.76 (0.45–1.30)
	US$100	26/64	41% (29–54)	US$2,000	35/64	55% (42–67)	0.72 (0.43–1.21)
	US$1,000	33/64	52% (39–64)	US$2,000	35/64	55% (42–67)	0.94 (0.60–1.48)
Halpern [47] A-20% risk of adverse effect	US$100	23/64	36% (24–49)	US$1,000	26/64	41% (29–54)	0.92 (0.30–1.70)
	US$100	23/64	36% (24–49)	US$2,000	29/64	45% (33–58)	0.80 (0.45–1.43)
	US$1,000	26/64	41% (29–54)	US$2,000	29/64	45% (33–58)	0.87 (0.50–1.51)
Halpern [47] A-30% risk of adverse effect	US$100	18/64	28% (18–41)	US$1,000	23/64	36% (24–49)	0.75 (0.37–1.53)
	US$100	18/64	28% (18–41)	US$2,000	25/64	39% (27–52)	0.69 (0.35–1.39)
	US$1,000	23/64	36% (24–49)	US$2,000	25/64	39% (27–52)	0.92 (0.50–1.70)
Halpern [47] B- 10% assigned to placebo	US$100	21/62	34% (22–47)	US$1,000	27/62	44% (31–57)	0.79 (0.43–1.45)
	US$100	21/62	34% (22–47)	US$2,000	28/62	45% (33–58)	0.70 (0.43–1.45)
	US$1,000	27/62	44% (31–57)	US$2,000	28/62	45% (33–58)	1.00 (0.58–1.73)
Halpern [47] B- 30% assigned to placebo	US$100	20/62	32% (21–45)	US$1,000	25/62	40% (28–54)	0.77 (0.40–1.48)
	US$100	20/62	32% (21–45)	US$2,000	26/62	42% (30–55)	0.77 (0.40–1.48)
	US$1,000	25/62	40% (28–54)	US$2,000	26/62	42% (30–55)	1.00 (0.56–1.80)
Halpern [47] B- 50% assigned to placebo	US$100	19/62	31% (20–44)	US$1,000	23/62	37% (25–50)	0.83 (0.42–1.64)
	US$100	19/62	31% (20–44)	US$2,000	27/62	44% (31–57)	0.71 (0.38–1.36)
	US$1,000	23/62	37% (25–50)	US$2,000	27/62	44% (31–57)	0.86 (0.48–1.54)

RR, experimental recruitment strategy/standard recruitment strategy. Used total number/number of experimental strategies to calculate RR, so that standard was not overrepresented. Halpern's study used each participant more than once.

a Studies showed a statistically significant difference in consent rates between recruitment strategies.

The Halpern study assessed the effect of variations in monetary incentives on the willingness to participate in a number of mock clinical studies (of varying trial designs that was mentioned earlier). Patients' willingness to participate increased as the payment level increased from US$100 to US$2,000 irrespective of the risk of adverse effect and risk of being assigned to placebo, although the difference was not statistically significant.

#### Methods of providing information

**Figure 6:**
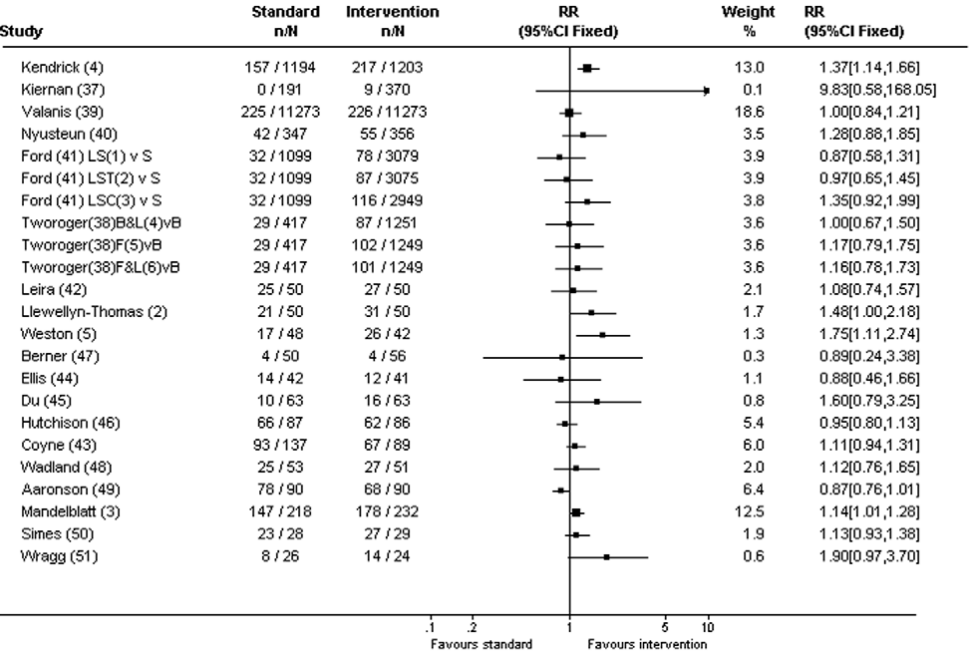
Consent rates for methods of providing information. RR, intervention recruitment strategy/standard recruitment strategy. Used total number/number of intervention strategies to calculate RR, so that the number of patients on standard strategies were not overrepresented; S, standard informed consent; B, bulk mailing; 1, enhanced recruitment letter and screening by African American interviewer; 2, enhanced recruitment letter, screening by African American interviewer and baseline information collected via telephone interview; 3, enhanced recruitment letter, screening by African American interviewer and church-based project sessions; 4, bulk mailing with letter; 5, first-class mailing; 6, first-class mailing with letter.

Nineteen recruitment trials compared different methods of providing information to participants, including how the information was presented and what information was provided (see Figure 6 and Table 6).

**Table 6:** Studies of methods of providing information.

Study	Standard Recruitment Strategy	*n/N*	Consent Rate (95% CI)	Experimental Recruitment Strategies	*n/N*	Consent Rate (95% CI)	RR (95% CI)
Kendrick [4]	Standard informed consent (mailing)	157/1,194	13% (11–15)	Additional home safety questionnaire	217/1,203	18% (16–20)	1.37 (1.14–1.66)^a^
Kiernan [28]	Standard informed consent (mailing of flyer)	0/191	0% (0–2)	Additional personal letter (combination of general letter+Hispanic specific letter)	9/370	2% (1–5)	9.83 (0.58–168.04)
Valanis [34]	Standard informed consent (mailing)	225/11,273	2% (2–6)	Advanced postcard 1 wk prior to mailing of recruitment packet	226/11,273	2% (2–2)	1.0 (0.84–1.21)
Nystuen [31]	Standard informed consent (mailing)	42/347	12% (9–16)	Additional reminder phone call for nonresponders	55/356	15% (12–19)	1.28 (0.88–1.85)
Ford [25]	Standard informed consent (mailing)+screening^b^	95/3,297	3% (2–4)	Enhanced recruitment letter+screening by African American interviewer	78/3,079	3% (2–3)	0.87 (0.58–1.31)
				Enhanced recruitment letter+screening by African American interviewer+baseline information collected via telephone interview	87/3,075	3% (2–3)	0.97 (0.65–1.45)
				Enhanced recruitment letter+screening by African American interviewer+church-based project sessions	116/2,949	4% (3–5)	1.35 (0.92–1.99)
Tworoger [37]	Bulk mailing no letters	86/1,250	7% (6–8)	Bulk mailing with letter	87/1,251	7% (6–9)	1.00 (0.67–1.50)
				First class mailing no letters	102/1,249	8% (7–10)	1.17 (0.79–1.75)
				First class mailing with letters	101/1,249	8% (7–10)	1.16 (0.78–1.73)
Leira [29]	Standard informed consent	25/50	50% (36–65)	Advanced notification with phone and fax	27/50	54% (39–68)	1.08 (0.74–1.57)
Llewellyn-Thomas [2]	Tape recording of trial information	21/50	42% (28–57)	Interactive computer program for participants	31/50	62% (47–75)	1.48 (1.00–2.18)^a^
Weston [5]	Standard informed consent	17/48	35% (22–51)	Additional video about the health condition	26/42	62% (46–76)	1.75 (1.11–2.74)^a^
Berner [45]	Standard informed consent (verbal)	4/50	7% (2–19)	Additional written cancer-specific information	4/56	7% (2–17)	0.89 (0.24–3.38)
Ellis [24]	Standard informed consent	14/42	33% (20–50)	Additional education booklet on trials	12/41	29% (16–46)	0.88 (0.46–1.66)
Du [39]	Standard informed consent	10/63	16% (8–27)	Additional video about clinical trials	16/63	25% (15–38)	1.60 (0.79–3.25)
Hutchison [38]	Standard informed consent	66/87	76% (66–84)	AVPI tool to explain about trials, video+DVD/CD	62/86	72% (61–81)	0.95 (0.80–1.13)
Coyne [48]	Standard informed consent	93/137	68% (59–76)	Easy-to-read consent statement	67/89	75% (65–84)	1.11(0.94–1.31)
Wadland [35]	Patients reading trial information	25/53	47% (33–61)	Study coordinator reading and explaining the study to patients	27/51	53% (39–67)	1.12 (0.76–1.65)
Aaronson [20]	Standard informed consent	78/90	87% (78–93)	Additional phone-based contact with oncology nurse	68/90	76% (65–84)	0.87 (0.76–1.01)
Mandelblatt [3]	Standard informed consent (brochure)	147/218	67% (61–74)	Additional brief educational session and discussion about the trial	178/232	77% (71–82)	1.14 (1.01–1.28)^a^
Simes [33]	Total disclosure	23/28	82% (63–94)	Individual approach	27/29	93% (77–99)	1.13 (0.93–1.38)
Wragg [44]	Explicit information^c^	8/26	31% (14–52)	Ambiguous information^d^	14/24	58% (37–78)	1.90 (0.97–3.70)

RR, experimental recruitment strategy/standard recruitment strategy. Used total number/number of experimental strategies to calculate RR, so that standard was not overrepresented.

a Studies showed a statistically significant difference in consent rates between recruitment strategies.

b Standard informed consent and screening (used total number/number of experimental strategies to calculate RR, so that standard was not overrepresented).

c Provides the current best estimates of effect of the experimental treatment.

d Emphasises the current state of uncertainty.

There were six recruitment trials that related to mailing of recruitment material for the clinical study. The methods used to enhance recruitment were the addition of: a questionnaire that focused on the health problem studied (Kendrick [32]); a personal letter inviting participation (Kiernan and Tworoger [33],[34]); use of bulk mailing or first class stamps (Tworoger [34]); an advanced postcard alerting recipients to look for the recruitment packet (Valanis [35]); a reminder phone call for nonresponders of mailed recruitment material (Nystuen [36]); and increasingly intensive interventions (for African Americans), which included a follow-up eligibility-screening phone call, an enhanced recruitment letter featuring a prominent African American man, recruitment by an African American member of the research team, and involvement of church-based project sessions (Ford [37]). Kendrick's addition of the questionnaire that focused on the health problem studied (RR 1.37, 95% CI 1.14–1.66) [32] was the only mailing strategy that increased the consent rate compared with standard mailing of recruitment material. The personal letter [33],[34] using bulk mail or first class mail [34], advanced postcard warning [35], and reminder phone calls [36] did not significantly increase consent rates (see Table 6).

Leira compared standard consent (being invited to participate in the clinical study when the investigators met the patient during helicopter retrievals) with advanced notification of the clinical study with telephone and faxing of informed consent documents prior to arrival of investigators in the helicopter [38]. The intention-to-treat analysis showed no statistical difference between the two recruitment strategies (RR 1.08, 95% CI 0.74–1.57), although 42% of the intervention group did not actually receive the intervention (fax and telephone call) because of technical and logistic reasons. Coyne compared an easy-to-read consent statement with standard consent [39] but showed no significant difference in consent rates (RR 1.11, 95% CI 0.94–1.31).

Three recruitment trials looked at increasing participants' understanding of the clinical trial process, which did not appear to affect recruitment [40]–[42]. Ellis compared standard informed consent with the addition of an educational booklet on clinical trials [40]. There was no difference in consent rates (unadjusted) between the two groups (RR 0.88, 95% CI 0.46–1.66). However, after adjusting for potential confounders (demographic variables, disease variables, preference for involvement in clinical decision making, anxiety, depression, and attitudes to clinical trials), participants receiving the educational booklets were significantly less likely to consent to clinical study participation (OR 0.22, 95% CI 0.04–1.0). Du compared standard care with the addition of a brief video about cancer clinical studies among patients with lung cancer [41]. Consent rates were not statistically different between the two groups. Hutchison compared standard care (where patients discuss clinical care and clinical study participation with the administration of a trial-specific information sheet and consent form) with the addition of an audiovisual patient information tool (with choice of video, CD-Rom, or DVD format), which addressed clinical trial information [42], with no difference in consent rates between the two groups (76% versus 72%, RR 0.95, 95% CI 0.80–1.13).

Three recruitment trials assessed strategies that aim to increase participants' understanding of their underlying condition. Llewellyn-Thomas compared tape recorded reading of clinical study information with an interactive computer program where participants (who were oncology patients receiving radiation therapy) were actively involved in the information search process [43]. The consent rate was higher for participants in the interactive group (RR 1.48, 95% CI 1.00–2.18). Weston compared standard informed consent with the addition of a video explaining trial information and the health problem studied [44]. The consent rate was higher in the video group when initially assessed (RR 1.75, 95% CI 1.11–2.74), but this did not reach statistical significance at 2 wk follow-up (not shown on Table 6). Berner's recruitment trial compared standard care (verbal communication) with the addition of patient information files containing clinical information on cancer specific to the patient [45]. There was no difference in the rate of recruitment to cancer trials in both groups (7% versus 7%, RR 0.89, 95% CI 0.24–3.38), although not all patients were eligible for clinical study enrolment.

Three recruitment trials compared standard consent with additional personal contact with research staff (a study coordinator reading and explaining the clinical study, Wadland [46]; additional phone-based contact with an oncology nurse, Aaronson [47]; and an additional educational session about the disease and risks and benefits of clinical study participation for an oncology prevention study, Mandelblatt [48]). There was no difference in consent rates between standard consent and the study coordinator reading and explaining the clinical study (RR 1.12, 95% CI 0.76–1.65) [46] or additional phone-based contact with the oncology nurse (RR 0.87, 95% CI 0.76–1.01) [47]. However there was higher consent for participants who attended the education session (RR 1.14, 95% CI 1.01–1.28) [48].

There were two recruitment trials assessing framing of recruitment information. In Simes' 1986 trial of recruitment for a cancer treatment study [49], total disclosure of information about the clinical study was compared with an individual approach where doctors informed patients about the clinical study in a manner they thought best. This study assessed both willingness to enrol in the clinical study and actual study participation. There were no differences in actual consent rates between the total disclosure and individual approach groups (RR 1.13, 95% CI 0.93–1.38). However, actual consent rates were higher than the stated willingness to participate in the clinical study (actual consent rates were 82% and 93% in the total disclosure and individual approach groups, respectively, compared with rates of 65% and 88%, respectively, for willingness to participate in the clinical study). Wragg compared framing of recruitment information explicitly (to provide the best current estimates of effect for the experimental treatment) with framing information ambiguously (to emphasise the uncertainty and relative costs and benefits of the experimental treatment) [50]. There was no difference in consent rates between the “ambiguously framed” group and the “explicitly framed” group (RR 1.90, 95% CI 0.97–3.70).

## Discussion

Trials of recruitment strategies have evaluated all steps in the recruitment process, including different methods of trial design, randomisation, provision of information, and recruiter differences. In this systematic review, we found that strategies that increased potential participants' awareness of the health problem being studied by engaging them in the learning process significantly increased consent rates (both for “real” and mock trials). These strategies included the addition of a questionnaire that focused on the health problem studied and additional educational sessions, videos, and interactive programs about the diseases studied [32],[43],[44],[48]. Strategies that increased understanding of the clinical trial process (e.g., provision of an educational booklet [40], video [41], or audiovisual patient information tool [42] on clinical trials or provision of an easy-to-read consent statement [39]) showed no evidence of improved recruitment. This finding suggests that it is increased education about the health problem being studied rather than education about the clinical trial process that increased trial participation. There were insufficient data to evaluate whether the effects of the different recruitment strategies were constant across all health conditions, but no there was no clear trend for these strategies to be context specific (see Table 1).

The recruitment trials on how recruitment information was provided (the technique of information presentation, how information was framed, who presented the information, and when the information was presented) did not show a difference between strategies, demonstrating that how or when the information was presented or who presented the information did not influence recruitment, but rather the information provided. A recent study (which was published after completion of our last search update) also showed that publicity about the trial did not increase recruitment [51].

Although a previous observational study showed that framing of recruitment information to emphasise uncertainty enhanced recruitment [52], when this was tested by the rigor of RCT methodology [49],[50], we found that framing did not appear to influence recruitment. Unexpectedly we found that the role of the recruiter also did not show evidence of influencing recruitment (although costs were higher for senior recruiters [28],[29]).

In our review, one recruitment trial identified that a noninferiority clinical study (with active treatment arms) had higher consent rates compared with a placebo-controlled clinical study. This finding is consistent with previous findings that patients preferred “trials with all active arms to placebo-controlled trials” [53]. Also, recruitment trials that compared standard placebo-controlled design with a nonblinded trial design demonstrated that patients were more willing to participate in a clinical study if they knew which treatment they were receiving when consenting, even if the treatment was randomly predetermined. These studies illustrate people's anxieties regarding the unknowns of clinical trial participation. Despite the higher consent rates for the nonblinded trial design, the differential loss to follow up in the two treatments arms of the nonblinded trial is likely to jeopardise validity of the results, as comparison of outcomes between the two treatment groups would be subject to selection bias. For example, patients may be more likely to drop out if they were unhappy with the treatment they were assigned. In the two included studies of nonblinded trial designs, there were higher drop outs in the active treatment arms compared with the placebo arms.

The inclusion of recruitment trials of recruitment to mock clinical studies enabled assessment of recruitment strategies, which for equity reasons would be difficult to compare (such as different randomisation designs, different monetary incentives). Some strategies may be acceptable when used in isolation, but inappropriate when more than one are used within the same clinical study: for example mock trials that tested the hypothesis that potential participants are more willing to participate in a study if they had an increased chance of receiving the experimental treatment is a strategy that has been adopted by many vaccine and other clinical studies in the belief that potential participants are more likely to participate if they believed they had a higher chance of receiving the (desirable) experimental treatment. However, we found that increasing the likelihood of receiving the experimental treatment [19] (or reducing the risk of receiving placebo) [14] did not appear to affect the consent rate, demonstrating that people's decisions for clinical study participation are not influenced by whether they are more or less likely to receive a particular treatment. Other strategies are more controversial: for example, the only consent strategy that appeared to affect the consent rate for a mock trial was “prerandomisation to standard drug” [18], where participants were given the standard drug and nonparticipants were given the experimental drug. Fewer people were willing to consent to this type of clinical study than to a clinical study of standard randomisation for all participants. It is unlikely that such a method could ethically be employed in a real situation. Monetary incentives appeared to increase consent compared to no monetary incentives [31], but the amount of money appeared to be less important [14].

As results of mock clinical studies are based on whether participants are willing to enrol in a clinical study (rather than whether they actually consented), extrapolation to real clinical studies may not be realistic. Stated “willingness to participate” and actual participation may also differ. In the recruitment trial comparing standard consent to the addition of a video explaining clinical trial information and the health problem studied for a mock clinical study, although statistically more participants from the video group were willing to enrol in the clinical study, this number became not statistically significant 2 wk later [44]. Conversely, in Sime's 1986 study [49], more participants actually consented to clinical study participation than had indicated willingness to participate, perhaps reflecting patients' deference to doctors' advice in the 1980s (when there was less emphasis on patient autonomy compared with today). It also showed the influence of the doctor on patient behaviour [53].

Although there have been two previous systematic reviews on strategies to enhance recruitment to research [9],[10], our study is the latest and has a more targeted and rigorous search method. We conducted a more comprehensive search (with inclusion of more databases than Watson's study [10]) and included earlier as well as later studies, and also studies of recruitment for mock trials to test recruitment strategies that would otherwise be difficult to compare for equity reasons. Our methods were also more rigorous (with two reviewers examining all titles, abstracts, and relevant papers) with an inclusion criteria targeting recruitment of participants for RCTs only (excluding studies about recruitment to observational studies, questionnaires, health promotional activities. and other health care interventions). We targeted recruitment to RCTs in which recruitment is more difficult because potential participants must consent to participation in research in which their treatment is unknown. The Mapstone study conducted in 2002 and published in 2007 [9] included recruitment for any type of research studies, and the Watson study [10], although targeting recruitment strategies used for RCTs, searched only from 1996 to 2004 with a limited number of electronic databases (without hand searching), using only the keywords “recruitment strategy” or “recruitment strategies.” Our study has identified more studies than the previous reviews (37 compared with 14 and 15 studies), and provides a better understanding of the factors that influence clinical RCT participation for potential participants. Although both previous studies highlighted effective and ineffective strategies, there was no attempt to examine the differences between successful and unsuccessful recruitment strategies.

Our findings are consistent with the health belief model that people are more likely to adopt a health behaviour (such as participation in a clinical study) if they perceive they are at risk of a significant health problem [54]. The importance of informing potential participants about the health problem being studied and engaging them in the learning process is not only educational and constructive, but is also likely to enhance clinical trial participation.

### Limitations

Because of major differences in recruitment methods, populations, and types of clinical studies that were recruiting as well as outcomes measured, we did not combine the results statistically in a meta-analysis. In many of the smaller recruitment trials, the failure to find a significant difference in consent rates could be related to the sample size (type II error). There may also be publication bias. However, as more than 70% (27/37) of the included studies had a nonsignificant result, we are hopeful that publication bias may be minimal. Given that the interventions we are considering are of noncommercial value we would suggest that publication bias may be less likely than for other interventions.

The majority of the included trials were conducted in developed countries, with a substantial proportion in the US. We acknowledge that developed countries' health systems may be very different from those of less-developed countries and hence the results of this systematic review may not be generalizable to other countries.

The main limitation of the study, due to the prolonged conduct of the study (from 2000 to 2009), was that the search strategy had to be modified with subsequent search updates owing to changes in MEDLINE Mesh heading definitions. Because of these changes (and the large number of titles and abstracts searched), the reason for exclusion of each study cannot be provided. The abstract of the first version of this systematic review (which included nonrandomised studies owing to the lack of randomised recruitment trials on the subject at the time) was published in conference proceedings in 2002 [11], and a later version that was limited to randomised studies was published in conference proceedings in 2007 [12].

### Conclusion

Our systematic review of recruitment strategies for enhancing participation in clinical RCTs has identified a number of effective and ineffective recruitment strategies. Grouped together, the statistically significant strategies either engaged participants in learning about the health problem being studied and its impact on their health or else informed participants of the treatment they have been randomised to receive (nonblinded trial design). However, as there was differential loss to follow up in the different treatment arms with nonblinded trial design, this trial design is likely to jeopardise the validity of the results. The use of monetary incentives may also increase recruitment, but as this was tested in a mock trial, and as another mock trial did not show any difference in consent rates between different amounts of monetary incentives, this finding needs to be interpreted with caution.

Future RCTs of recruitment strategies that engaged participants in the learning process using various methods of delivering the recruitment material compared with standard recruitment may confirm the effectiveness of this concept. This research may be particularly useful for testing strategies that expose large number of potential participants to recruitment information such as interactive internet strategies.

## Supporting Information

Text S1 PRISMA checklist.
(0.07 MB DOC)

## Abbreviations

CI: confidence interval
RCT: randomised controlled trial
RR: relative risk
